# Hypoxia-inducible microRNA-224 promotes the cell growth, migration and invasion by directly targeting RASSF8 in gastric cancer

**DOI:** 10.1186/s12943-017-0603-1

**Published:** 2017-02-07

**Authors:** Chuan He, Libo Wang, Jiantao Zhang, Hong Xu

**Affiliations:** 1 Department of Gastroenterology, First Hospital of Jilin University, No.71 Xinmin Street, Changchun, Jilin 130021 People’s Republic of China; 2 Department of Colorectal and anal surgery, First Hospital of Jilin University, Changchun, Jilin 130021 People’s Republic of China

**Keywords:** microRNA (miRNA), miR-224, Gastric cancer, RASSF8, Hypoxia-inducible factor-1α (HIF-1α)

## Abstract

**Background:**

Hypoxia plays an important role in the development of various cancers. MicroRNAs (miRNAs) act as post-transcriptional regulators of gene expression and modulate the tumorigenesis, including gastric cancer. However, the roles and molecular mechanism of miR-224 in gastric cancer under hypoxia remain poorly understood.

**Method:**

Real-time PCR and Northern blot assay were used to examine the effects of hypoxia and HIF-1α on miR-224 expression. Luciferase and ChIP assays were performed to determine whether miR-224 was a transcriptional target of HIF-1α. Then MTT, colony formation, in vitro scratch and invasion assays were used to detect the effects of miR-224 on cell growth, migration and invasion under hypoxia, as well as the in vivo animal study. Luciferase assay and Western blot were performed to validate the targets of miR-224. Functional studies were performed to determine the roles of RASSF8 as that of miR-224 under hypoxia. The effects of RASSF8 knockdown on the transcriptional activity and translocation of NF-κB were investigated using Luciferase assay and Western blot, respectively. Finally, the expression levels of miR-224 and RASSF8 were detected using real-time PCR in gastric cancer tissues as well as lymph node metastasis tissues.

**Results:**

We demonstrated that miR-224 was upregulated by hypoxia and HIF-1α. HIF-1α affected miR-224 expression at the transcriptional level. MiR-224 inhibition suppressed cell growth, migration and invasion induced by hypoxia, while miR-224 overexpression resulted in opposite effects. MiR-224 inhibition also suppressed tumor growth in vivo. We then validated that RASSF8 was a direct target of miR-224. RASSF8 overexpression inhibited cell growth and invasion, while RASSF8 knockdown ameliorated the inhibitory effects of miR-224 inhibition on cell growth and invasion. Furthermore, we found that RASSF8 knockdown enhanced the transcriptional activity of NF-κB and p65 translocation, while RASSF8 overexpression resulted in opposite effects. Inhibition of NF-κB activity by PDTC attenuated the effects of RASSF8 knockdown on cell proliferation and invasion. Finally, miR-224 was upregulated in both gastric cancer tissues and lymph node metastasis positive tissues, while RASSF8 expression was opposite to that of miR-224.

**Conclusion:**

These results indicate that hypoxia-inducible miR-224 promotes gastric cancer cell growth, migration and invasion by downregulating RASSF8 and acts as an oncogene, implying that inhibition of miR-224 may have potential as a therapeutic target for patients with hypoxic gastric tumors.

## Background

Gastric cancer is the most common epithelial malignancy and the second leading cause of cancer-related death worldwide [1]. Patients with advanced gastric cancer usually have a poor prognosis despite the combined therapy including gastriectomy, chemotherapy and radiotherapy. Therefore, better understanding the pathogenesis of gastric cancer and exploring novel therapeutic targets are urgent.

Hypoxia is a common feature of various cancers. It may cause the cells to acquire more aggressive phenotypes, such as cell migration, invasion, growth and metastasis, by changing genetic programs which can facilitate cellular adaptation to hypoxic stress [2]. Hypoxia-inducible factor 1 (HIF-1), an important transcriptional regulator, is an essential mediator that plays crucial roles in the cell response to hypoxia by modulating hypoxic gene expression [3, 4]. HIF-1 consists a hypoxia-sensitive subunit HIF-1α and a constitutively expressed subunit HIF-1β. Under normoxic condition, HIF-1α is degraded via the recruitment of an ubiquitin-protein ligase. Whereas in hypoxic condition, HIF-1α is activated via the decreased hydroxylation by PHD [5]. Accumulating evidence shows that microRNAs (miRNAs) play important roles in the articulated molecular mechanism triggered by hypoxia [6].

MiRNAs are a conserved family of small non-coding RNA molecules that act as important regulators of gene expression at the post-transcriptional level [7]. They can bind to the 3′untranslated region (3′UTR) of target genes, resulting in the target mRNA degradation or translational repression [8, 9]. MiRNAs are reported to be involved in diverse biological processes, such as cell proliferation, apoptosis and death [10]. Previous studies demonstrate that miRNAs are important regulators of cell response to hypoxia. For instance, miR-210 is induced by HIF-1α under hypoxia and acts as an independent prognostic biomarker in breast cancer [11]. HIF-1α-inducible miR-382 promotes angiogenesis and acts as an oncogene by directly targeting PTEN in gastric cancer under hypoxia [12]. A previous study shows that HIF-1α and hypoxia can upregulate miR-224 in melanoma cell lines [13] and primary human trophoblasts [14]. MiR-224 is reported to contribute to cell invasion and metastasis in human breast cancer cells [15] and human hepatocellular carcinoma [16]. However, the potential roles and molecular mechanism of miR-224 remain poorly understood in human gastric cancer exposed to hypoxia.

In this study, we found that miR-224 was induced by hypoxia and HIF-1α at the transcriptional level. MiR-224 inhibition suppressed the cell growth, migration and invasion induced by hypoxia, while miR-224 overexpression had opposite effects. RASSF8 was validated to be a direct target of miR-224 and mediated the effects of miR-224. RASSF8 knockdown enhanced NF-κB transcriptional activity and p65 translocation, while the inhibition of NF-κB ameliorated the roles of RASSF8 knockdown in cell proliferation and invasion. MiR-224 and RASSF8 were inversely expressed in gastric cancer tissues.

## Methods

### Clinical samples, cell culture and transfections

Gastric cancer tissue samples were acquired from 29 patients with informed consent in the department of Gastroenterology of the First Hospital of Jilin University. All tissues were frozen in liquid nitrogen and stored at −80 °C. The clinicopathological information for all patients were shown in Table 1.

**Table 1:** The relationship between miR-224 level and the clinicopathological features of 29 gastric cancer patients

Categories	Number of patients (*n* = 29)	Relative miR-224 level	*P* value
Age (year)			
< 60	15	0.37 ± 0.08	0.230
>= 60	14	0.32 ± 0.11	
Gender			
Male	14	0.36 ± 0.10	0.403
Female	15	0.34 ± 0.12	
Tumor size (cm)			
< 5 cm	13	0.29 ± 0.10	0.015
>= 5 cm	16	0.38 ± 0.07	
Depth			
T1	12	0.25 ± 0.07	0.001
T2–T4	17	0.40 ± 0.07	
Lymph node metastasis			
Absent	13	0.25 ± 0.06	0.001
Present	16	0.42 ± 0.05	
TNM stage			
I, II	11	0.28 ± 0.05	0.005
III, IV	18	0.38 ± 0.11	

Human gastric cancer cell lines SGC-7901 and MGC-803 were cultured in RPMI 1640 medium (Invitrogen, USA) supplemented with 10% fetal bovine serum (FBS) and 1% penicillin/streptomycin (100 U/ml and 100 μg/ml, respectively). The cells were maintained in a humidified atmosphere with 5% CO_2_ and 20% O_2_ at 37 °C, which was referred as the normoxic condition. For hypoxia, the cells were maintained with 5% CO_2_, 1% O_2_ and 94% N_2_ in a hypoxic chamber (Invivo200, UK). The cell transfections were performed using Lipofectamine™ 2000 reagent (Invitrogen, USA) according to the manufacturer’s instructions.

### RNA isolation and real-time PCR

RNAs were extracted using Trizol Reagent (Qiagen, USA) from the transfected cells exposed to hypoxia or normoxic condition according to the manufacturer’s protocols. cDNA was synthesized from 500 ng of RNAs using M-MLV reverse transcriptase (Promega) and specific miR-224 reverse transcription primer. Real-time PCR was performed using SYBR Premix EX Taq (TaKaRa) and specific miR-224 primers on a 7900HT Fast Real-Time System (Applied Biosystems) according to the manufacturer’s instructions. The PCR was carried out according to following procedures: 95 °C 5 min, followed by 40 cycles of 95 °C 1 min, 56 °C 30 s and 72 °C 30 s. U6 snRNA was used as an internal control to normalize miR-224 expression. The primers used in reverse transcription and real-time PCR were listed as follows: miR-224 reverse transcription primer: 5′ CTTGCATCACCAGAGAACGAACGGAACC 3′; U6 reverse transcription primer: 5′ AAAATATGGAACGCTTCACGAATTTG 3′. MiR-224 forward primer: 5′ GCGAGGTCAAGTCACTAGTGGT 3′; miR-224 reverse primer: 5′ CGAGAAGCTTGCATCACCAGAGAACG 3′; U6 snRNA forward primer: 5′ CTCGCTTCGGCAGCACATATACT 3′; U6 snRNA reverse primer: 5′ ACGCTTCACGAATTTGCGTGTC 3′; RASSF8 sense primer: 5′AAGTATGGGTGGATGGAGTTCAG 3′; RASSF8 antisense primer: 5′ ATGAGGTGCTAAGTGTCTTTCAG 3′; β-actin sense primer: 5′ TGGCACCCAGCACAATGAA 3′; β-actin antisense primer: 5′ TAAGTCATAGTCCGCCTAGAAGCA 3′.

### Western blot assay

The cells treated under hypoxia or normoxia were harvested and lysed using RIPA lysis buffer (50 mM Tris-HCl, pH 8.8, 150 mM NaCl, 1% NP-40, 1% sodium deoxycholate, 0.1% SDS). Protein concentration was measured using a BCA protein assay kit. Fifty microgram of protein samples were resolved on 10% SDS-PAGE gels and then transferred to the PVDF membrane. The membrane was incubated with 5% milk in TBST buffer, followed by incubation with the primary antibodies. Mouse polyclonal to RASSF8 antibody and rabbit polyclonal to NF-κB p65 antibody were used as the primary antibodies purchased from Abcam Company (1:1000 dilution). HRP-conjugated goat anti-rabbit or mouse antibody was used as the secondary antibody (1:5000). The bands were visualized using enhanced chemiluminescence (ECL) detection kit according to the manufacturer’s instructions. GAPDH was employed as a loading control.

### Northern blotting assay

MiRNAs were isolated from treated cells using mirVana miRNA Isolation Kit (Ambion) according to the manufacturer’s instructions. RNA concentration was measured and then subjected to Northern blotting assay according to the procedures as previously described [17]. U6 snRNA was used as an internal control.

### MTT (3-(4,5-dimethyl-2-thiazolyl)-2,5-diphenyl-2-H-tetrazolium bromide) assay

Cell viability was measured using MTT assay according to the manufacturer’s protocols. Briefly, the transfected cells under normoxic or hypoxic conditions were incubated with MTT once the cells were adhesive to the plates. After incubation for 24, 48 and 72 h, the medium was replaced and the cells were treated with DMSO for 10 min, followed by the measurement with a spectrophotometer on 570 nm (A_570 nm_).

### Colony formation assay

The transfected cells were seeded in 12-well plates under normoxic or hypoxic conditions. The medium was refreshed every 3 days for approximately 10 days when most of the colony contained more than 50 cells. The colony was fixed, stained with 1% crystal violet and finally counted.

### In vitro scratch assay (cell migration assay)

When the transfected cells reached approximately 80% confluence in 48-well plates, a 200 μl pathogen-free tip was used to scratch the cells. The cells were then washed with PBS and cultured in RPMI 1640 medium with 1% FBS. The scratch was taken pictures at different time points under the microscope. The cell migration rate = (width at 0 h–width at different time points)/width at 0 h.

### Cell invasion assay

The transfected cells (2.5 × 10^4^ cells) were seeded in serum-free medium in modified Boyden chamber coated with Matrigel. The lower chamber contained RPMI 1640 with 10% FBS. After the cells invaded for 20 h, non-invading cells were scraped with cotton tips. The invading cells on the underside of the chambers were fixed, stained and counted under a microscope.

### Luciferase reporter assay

The 3′UTR of RASSF8 mRNA containing miR-224 binding sites was PCR-amplified and inserted downstream of a luciferase reporter gene in the pmirGLO vector. In addition, a mutant construct containing mutations within the binding sites was generated using the QuikChange® site-directed mutagenesis kit (Stratagene, USA) according to the manufacturer’s instructions. The cells were co-transfected with miR-224 mimics and wild-type or mutant luciferase reporter constructs, or transfected with wild-type or mutant luciferase reporter constructs under hypoxic condition. At 24 h after transfection, luciferase intensity was determined using the Dual-Luciferase Reporter Assay System (Promega) according to the manufacturer’s instructions. Renilla luciferase intensity was normalized to firefly luciferase intensity.

As for the binding of HIF-1α to miR-224 promoter, miR-224 promoter was predicted using Promoter 2.0 prediction server, cloned and inserted upstream of a luciferase open reading frame in the pGL3 promoter vector. A mutation within the binding sites between HIF-1α and miR-224 was generated using a QuikChange® site-directed mutagenesis kit (Stratagene, USA) according to the manufacturer’s recommendations. The luciferase activity was determined using the Dual-Luciferase Reporter Assay System (Promega) according to the manufacturer’s protocol. The pTK-luc (Renilla) vector was co-transfected with above constructs and served as a spiked-in control.

### Chromatin immunoprecipitation (ChIP) assay

ChIP assay was performed to determine the interaction between HIF-1α and miR-224 promoter using the ChIP assay kit (Merck Millipore) according to the manufacturer’s instructions. Gastric cancer cells were incubated under hypoxic or normoxic condition before harvested. Anti-HIF-1α antibody (Abcam, USA) was used to precipitate the DNA fragment. PCR was performed to analyze the binding of HIF-1α to the promoter of miR-224. The PCR primers were ccatcacttccctcagtggt and cccttgacttttccccactt. PCR products were analyzed by gel electrophoresis on a 1.5% agarose gel.

### In vivo animal study

The animal experiments were approved by the Institutional Animal Care and Use Committee of First Hospital of Jilin University and performed in accord with the institutional and NIH guidelines. Briefly, 10^6^ gastric cancer cells transfected with miR-224 antagomir or control were injected subcutaneously into the flanks of 6 young athymic nude mice (6–8 weeks old). Tumor volumes were measured every 5 days at 5 days after injection until day 25 when the mice were sacrificed. The volume was calculated by measuring the length (L) and width (W) of the xenografts. Xenograft volume = (L^2^ × W)/2.

### Detection of NF-κB transcription activity and p65 translocation

The cells were treated with a NF-κB luciferase reporter plasmid (Sigma, USA). The pTK-luc (Renilla) vector was transfected as a spiked-in control. The luciferase intensity was determined using the Dual-Luciferase Reporter Assay System (Promega) according to the manufacturer’s protocol.

The gastric cancer cells were subjected to subcellular fractionation using the NE-PER^TM^ Cytoplasmic and Nuclear Extraction reagents (Thermo) according to the manufacturer’s instructions. The efficiency of fractionation was examined by Western blotting assay using antibodies against LaminB1 (the nuclear control) and GAPDH (the cytosolic control).

### Statistical analysis

The data were expressed as mean ± standard deviation (SD) and obtained from three independent experiments. The difference between two groups was analyzed using two-tailed Students’ *t*-test. The relationship between miR-224 and RASSF8 was analyzed using Pearson correlation analysis. The analysis was performed using Graphpad Prism 5 project. The value of *P* < 0.05 was considered statistically significant.

## Results

### MiR-224 is hypoxia-responsive and upregulated by HIF-1α in gastric cancer cells

**Fig. 1:**
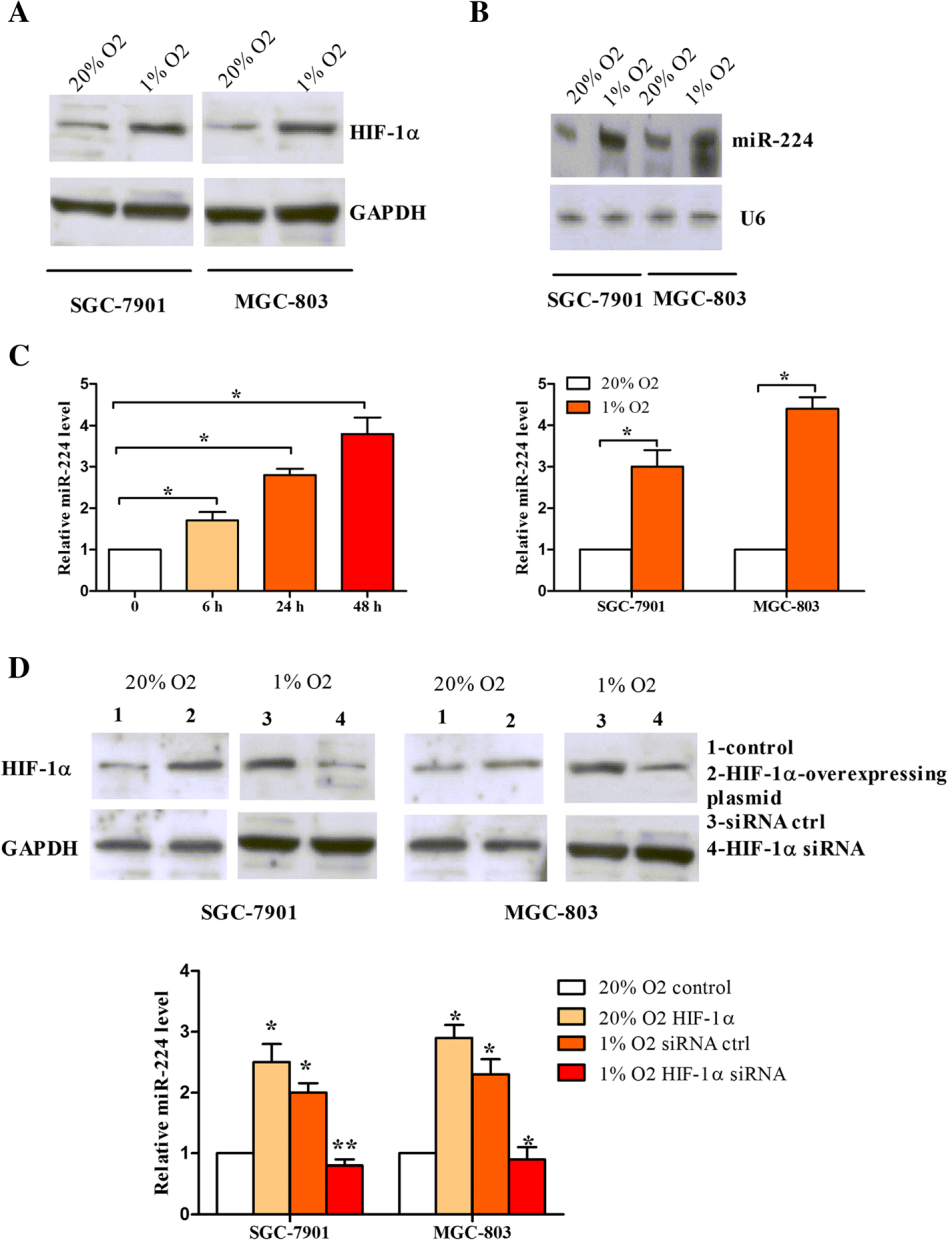
MiR-224 is hypoxia-responsive and upregulated by HIF-1α in gastric cancer cells. **a** HIF-1α expression was analyzed by Western blot in SGC-7901 and MGC-803 cells under hypoxia and normoxic condition. GAPDH was employed as a loading control. **b** MiR-224 expression was analyzed by Northern blotting in SGC-7901 and MGC-803 cells under hypoxia or normoxic condition. U6 snRNA was used as an internal control. The graph represented the relatively quantitative expression of miR-224. **c** MiR-224 expression was analyzed by real-time PCR in SGC-7901 cells exposed to hypoxia for 6, 24 and 48 h. U6 snRNA was used as an internal control. **d** SGC-7901 and MGC-803 cells were transfected with either HIF-1α overexpressing plasmid or HIF-1α siRNA or controls, and then subjected to Western blot or real-time PCR to determine HIF-1α or miR-224 expression levels. *P < 0.05, **P < 0.01

To investigate miR-224 expression in response to hypoxia, MGC-803 and SGC-7901 cells were incubated in hypoxic (1% O_2_) or normoxic (20% O_2_) conditions. We first detected HIF-1α expression using Western blotting assay, which is a positive indicator of hypoxia. As shown in Fig. 1a, HIF-1α protein level was upregulated under hypoxia. Moreover, we found that miR-224 expression was increased after the cells exposed to hypoxia (Fig. 1b). The cells were then exposed to hypoxia for 6, 24, and 48 h, and miR-224 expression was increased in a time-dependent manner, suggesting that miR-224 was upregulated substantially under subchronic hypoxia (Fig. 1c). We next determined whether the upregulation of miR-224 was related to HIF-1α. As shown in Fig. 1d, cells treated with HIF-1α-overexpressing plasmid had a higher level of miR-224 under normoxia, while silencing of HIF-1α resulted in a decrease in miR-224 expression under hypoxia. Taken together, these data indicate that the strong induction of miR-224 by hypoxia is HIF-1α-dependent.

### HIF-1α upregulates miR-224 expression at the transcriptional level

**Fig. 2:**
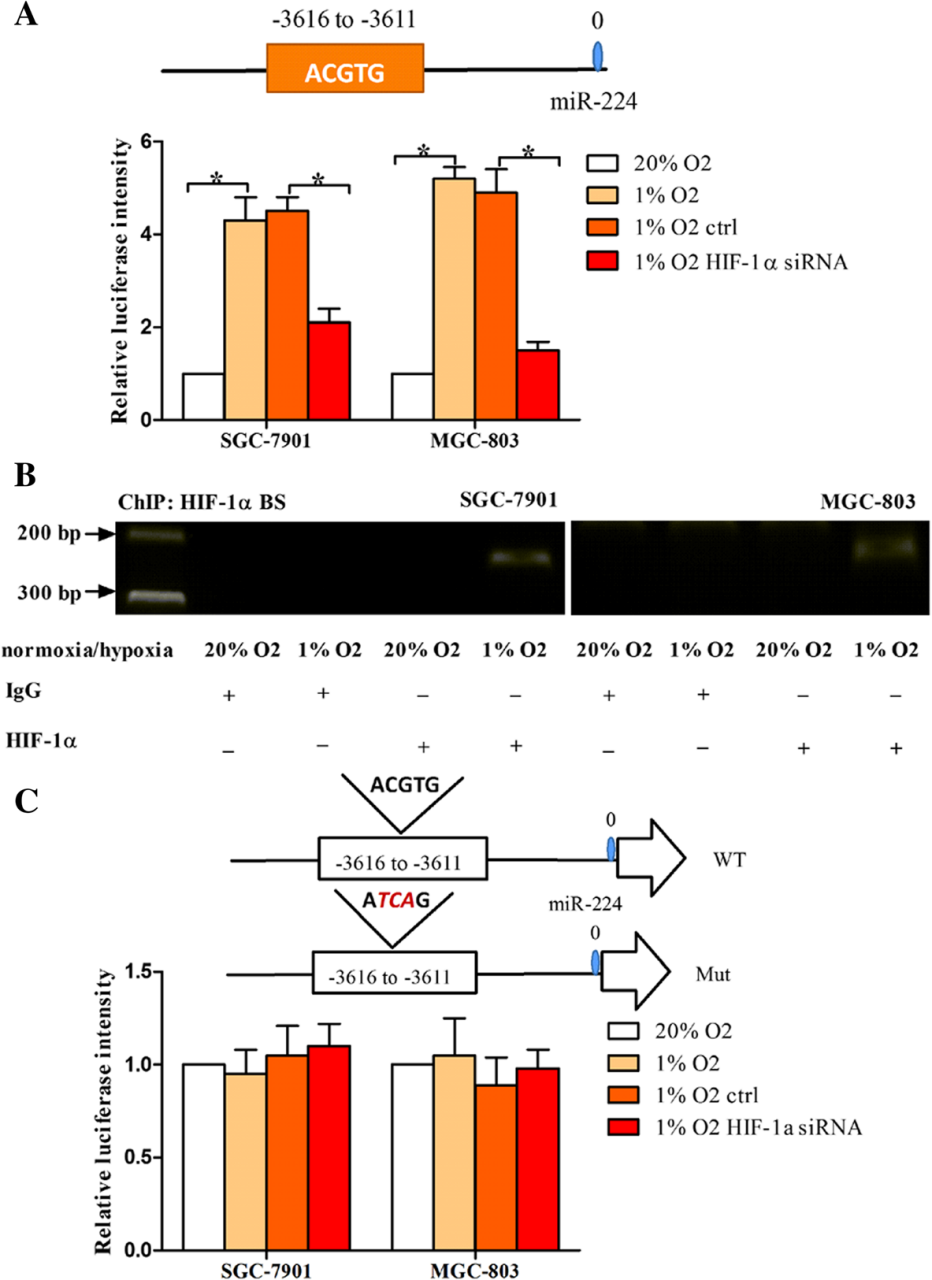
HIF-1α upregulates miR-224 expression at the transcriptional level. **a** There was a putative hypoxia response element (HRE) in the region of miR-224 promoter. MiR-224 promoter containing the HRE was cloned upstream of a luciferase reporter gene. SGC-7901 and MGC-803 cells were transfected with the above luciferase construct under hypoxia or normoxic condition, and the luciferase intensity of miR-224 promoter was determined. In addition, the cells were co-transfected the above construct and HIF-1α siRNA or si-control under hypoxia, and subjected to luciferase assay. **b** SGC-7901 and MGC-803 cells were exposed to hypoxia or normoxic condition and harvested for ChIP assay. Chromatin-bound DNA was precipitated with the antibody against HIF-1α. Anti-IgG antibody was used as a negative control. **c** Schematic representation of HIF-1α binding sites in miR-224 promoter, together with a mutation generated within the binding sites. The mutated binding sites were cloned upstream of the luciferase reporter gene. The cells were transfected with the mutant constructs under hypoxia or normoxia and subjected to luciferase assay. In addition, the cells were co-transfected the mutant construct and HIF-1α siRNA or si-control under hypoxia, and subjected to luciferase assay. *P < 0.05

Considering that HIF-1α is a transcription factor, we aimed to identify whether HIF-1α regulated miR-224 expression at the transcriptional level. A previous study shows that the HRE (hypoxia response element) of all hypoxia regulated genes contains the core sequence (A/G) CGTG, mostly ACGTG [18]. We searched for the potential HREs in the region upstream of pre-miR-224 sequence and found this core sequence (ACGTG) was in the region approximately 3.5 kb upstream of miR-224 precursor. We then cloned miR-224 promoter containing this core sequence in a luciferase reporter vector (pGL3). As shown in Fig. 2a, we found the luciferase intensity of miR-224 promoter was increased under hypoxia compared to normoxia. However, when HIF-1α was knockdown under hypoxia, miR-224 promoter activity was significantly decreased. Similar results were shown in MGC-803 and SGC-7901 cells. To identify the direct interaction between HIF-1α and miR-224 promoter, ChIP assay was performed under normoxic or hypoxic conditions (Fig. 2b). The results demonstrated that the protein/DNA complexes precipitated with anti-HIF-1α antibody resulted in a specific PCR product flanking the core sequence under hypoxia. We finally validated whether HIF-1α upregulated miR-224 expression through binding to the core sequence. A mutation was generated within HIF-1α binding sites on miR-224 promoter and we found that neither hypoxia nor HIF-1α knockdown affected miR-224 promoter activity (Fig. 2c). These results indicate that HIF-1α enhances miR-224 transcript levels via directly binding to the HRE in miR-224 promoter region.

### Inhibition of miR-224 suppresses cell growth, migration and invasion under hypoxia

**Fig. 3:**
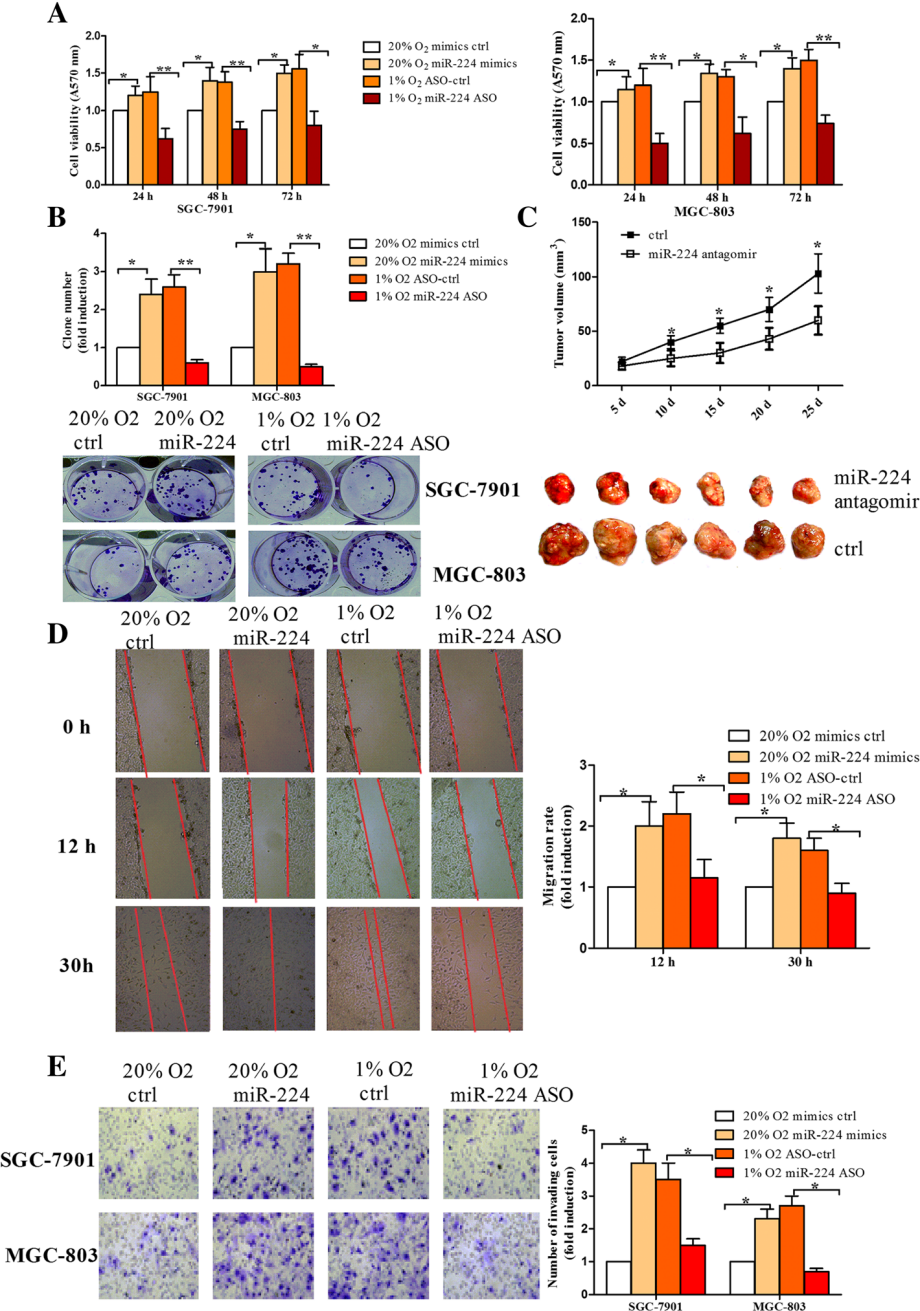
Inhibition of miR-224 suppresses the cell proliferation, tumor growth, cell migration and invasion induced by hypoxia in gastric cancer cells. **a** MTT assay: SGC-7901 and MGC-803 cells were transfected with miR-224 mimics under normoxia or miR-224 ASO under hypoxia, together with controls. Cell viability was measured by MTT assay at 24, 48 and 72 h after transfection. **b** Colony formation assay: the transfected cells as (**a**) were seeded in 12-well plates and medium was changed every 3 days until most colonies contained more than 50 cells. The pictures showed the colonies stained by crystal violet. **c** SGC-7901 cells transfected with miR-224 antagomir or controls were injected subcutaneously into the flanks of 6 young athymic nude mice, and tumor volumes were measured every 5 days until day 25 when the mice were sacrificed. The pictures showed the xenografts when the mice were sacrificed. **d** In vitro scratch assay (cell migration assay): When the confluence of the transfected cells reached approximately 80%, a scratch was generated with a 200 μl tip and the cells were incubated with 1% FBS-containing medium. The wound was taken pictures at different time points. **e** Cell invasion assay: The transfected cells were (2.5 × 104 cells) seeded in serum-free medium in a Matrigel-coated chamber, while the lower chamber was incubated with 10%-FBS containing medium. The cells were invaded for 20 h under hypoxia or normoxia. Non-invading cells were scraped with a cotton tips and invading cells were fixed and stained with crystal violet, followed by counted. *P < 0.05, **P < 0.01

Hypoxia has been associated with tumor aggressiveness because of its promoting roles in cell growth, migration and invasion [2]. Therefore, we tested whether hypoxia inducible miR-224 was involved in the above phenotypes. As shown in Fig. 3a, miR-224 overexpression increased SGC-7901 and MGC-803 cell viability under normoxic condition, while miR-224 inhibition resulted in a decreased in cell viability under hypoxia. In line with the effects of miR-224 on cell viability, we found that miR-224 promoted the colony-forming ability, while miR-224 inhibition suppressed the colony number induced by hypoxia (Fig. 3b). Furthermore, in vivo study showed that xenografts tumor masses made of miR-224 antagomir-treated cells had smaller volumes than xenografts made of control-treated cells (Fig. 3c). Taken together, the results indicate that miR-224 inhibition suppresses hypoxia-induced gastric cancer growth in vitro and in vivo.

We next investigated the effects of miR-224 on cell migration and invasion under hypoxic condition. As shown in Fig. 3d, miR-224 overexpression contributed to migration rate of MGC-803 cells under normoxia, while miR-224 inhibition suppressed the migration rate under hypoxia. Similarly, miR-224 inhibition suppressed hypoxia-induced cell invasion in MGC-803 and SGC-7901 cells (Fig. 3e).

### RASSF8 is a direct target of miR-224 in gastric cancer

**Fig. 4:**
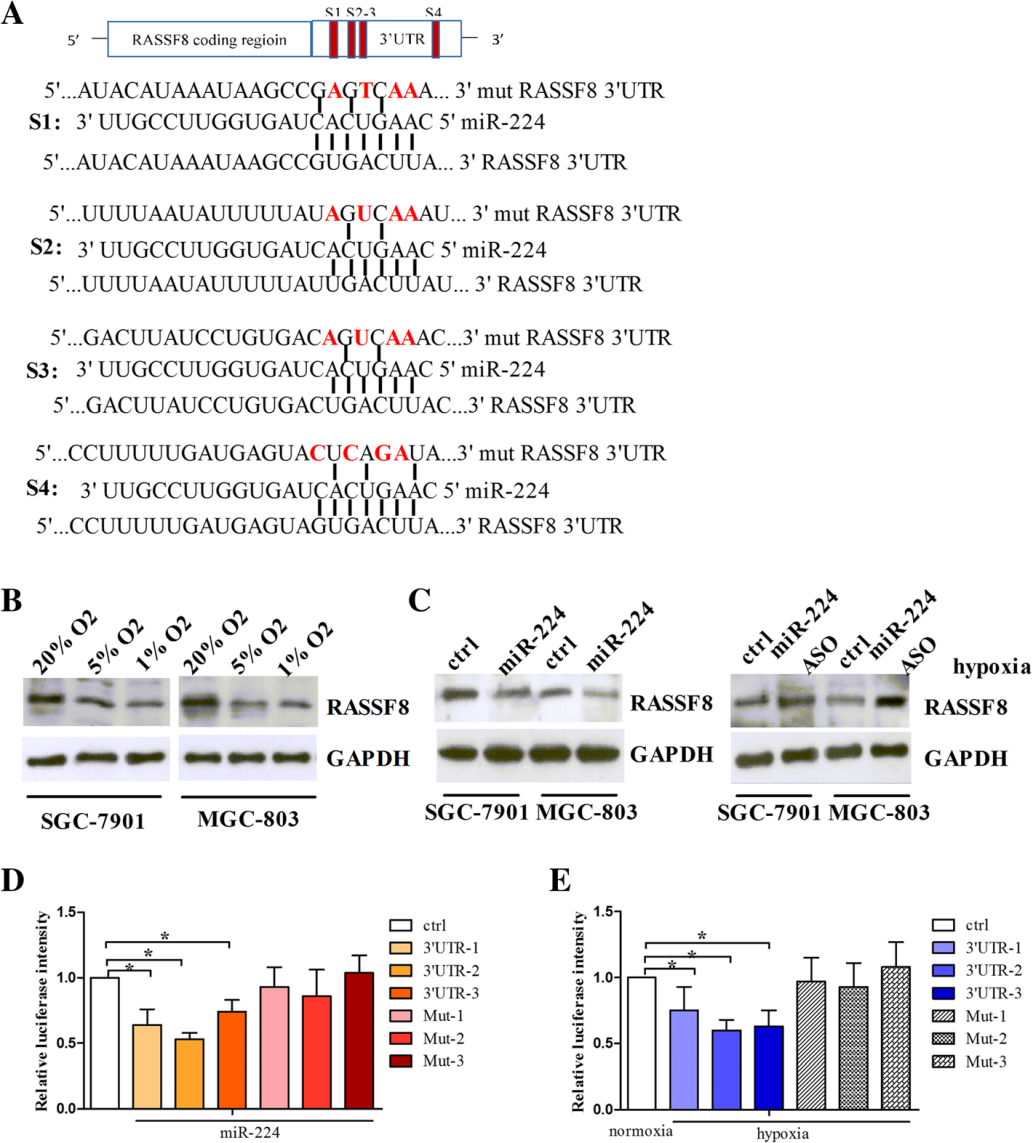
RASSF8 is a direct target of miR-224 in gastric cancer cells. **a** Sequence alignment of miR-224 and RASSF8 3′UTR. There were four putative binding sites on the RASSF8 3′UTR. The red bases represented the mutations. **b** RASSF8 protein levels were analyzed by Western blot in SGC-7901 and MGC-803 cells exposed to normoxia, 5% O2 and 1% O2. **c** SGC-7901 and MGC-803 cells were transfected with miR-224 or miR-224 ASO under normoxia or hypoxia, and then subjected to Western blot to examine RASSF8 protein levels. GAPDH was used as a loading control. **d** Luciferase assay: RASSF8 3′UTR containing miR-224 binding sites or mutant binding sites were cloned downstream of a luciferase reporter gene. The cells were co-transfected with miR-224 and wild-type or mutant luciferase constructs, together with controls, and then subjected to luciferase assay. **e** The cells transfected with wild-type or mutant luciferase constructs were exposed to hypoxia or normoxia, and then subjected to luciferase assay. *P < 0.05

We used algorithmic programs (miRanda, TargtScan and Pictar) to predict the potential targets of miR-224. Among the candidate targets, RASSF8 (RAS association domain family member 8) was selected for further study. As shown in Fig. 4a, there were four binding sites for miR-224 on RASSF8 3′UTR. Considering that miR-224 was upregulated by hypoxia, we tried to test the roles of hypoxia in FASSF8 expression. We found that RASSF8 expression was decreased under hypoxia in SGC-7901 and MGC-803 cells (Fig. 4b). Moreover, miR-224 suppressed RASSF8 protein level, while miR-224 inhibition resulted in an increase in RASSF8 protein expression under hypoxia (Fig. 4c). These results imply that RASSF8 may be a target for miR-224. We next cloned RASSF8 3′UTR containing the binding sites (Site 1, Site 2, Site 3 and Site 4) downstream of a luciferase reporter gene in a pmirGLO vector, named 3′UTR-1, 3′UTR-2 and 3′UTR-3 (site 2 and site 3 were cloned in a vector, named 3′UTR-2). As shown in Fig. 4d, we found that miR-224 overexpression reduced the luciferase intensity controlled by RASSF8 3′UTR. To confirm this result, several mutations were generated within the four binding sites and the mutant luciferase constructs were named Mut-1, Mut-2 and Mut-3 (Fig. 4a). Luciferase assay indicated that miR-224 did not inhibit the luciferase activity of the mutants (Fig. 4d). Similarly, under hypoxic condition, the luciferase intensity of RASSF8 3′UTR was decreased, while the mutant luciferase constructs were resistant to the hypoxic suppression of RASSF8 3′UTR intensity (Fig. 4e). Taken together, these data indicate that RASSF8 is a direct target of miR-224.

**Fig. 5:**
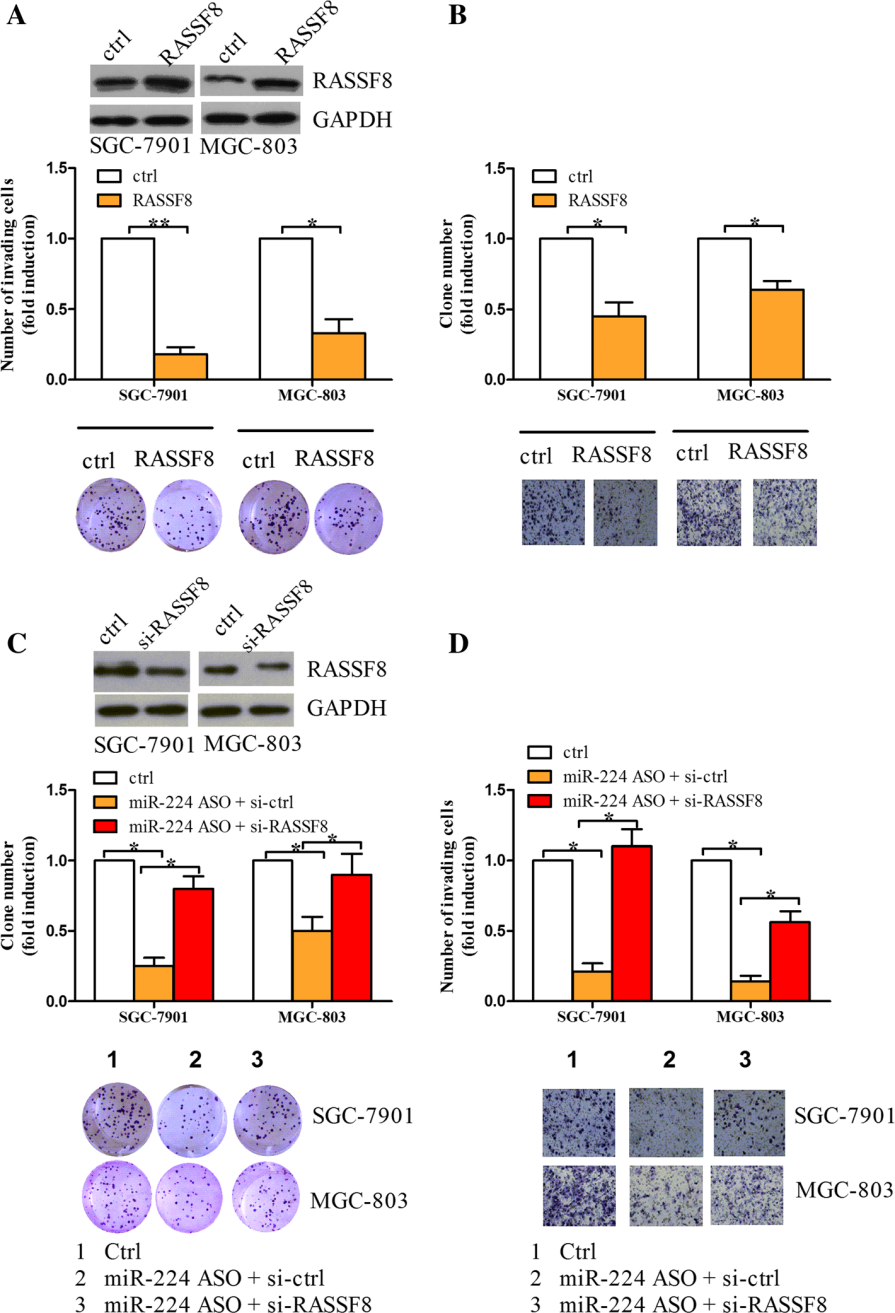
RASSF8 mediates the roles of miR-224 in cell growth and invasion. **a** The cells transfected with RASSF8-overexpressing plasmid or control were subjected to Western blot to determine RASSF8 expression. Colony formation assay was performed in the above cells to detect the roles of RASSF8 in cell proliferation. **b** Cell invasion assay: The above cells were (2.5 × 104 cells) seeded in serum-free medium in a Matrigel-coated chamber, while the lower chamber was incubated with 10%-FBS containing medium. The cells were invaded for 20 h. Non-invading cells were scraped with a cotton tips and invading cells were fixed and stained with crystal violet, followed by counted. **c**–**d** The cells were co-transfected with miR-224 ASO and RASSF8 siRNA under hypoxia, together with controls, and then subjected to colony formation assay (**c**) and cell invasion assay (**d**). Western blot assay showed that expression of RASSF8 in the above cells. *P < 0.05, **P < 0.01

To determine whether RASSF8 is a functional target of miR-224 in gastric cancer, we examined the roles of RASSF8 in the cell growth and invasion under hypoxia. We found that RASSF8 overexpression resulted in a decrease in the number of colonies and invasive cells in SGC-7901 and MGC-803 cells compared to control groups (Fig. 5a–b). Next, the cells were co-treated with miR-224 inhibitor and RASSF8 siRNA, together with control groups. As shown in Fig. 5c–d, RASSF8 knockdown restored the cell growth and invasion that were inhibited by miR-224 inhibitor under hypoxic condition. MGC-803 and SGC-7901 cells had similar results. These data suggest that miR-224 induced by hypoxia downregulates RASSF8 and RASSF8 is an important mediator of miR-224-induced functions in gastric cancer cells.

### RASSF8 modulates NF-κB transcriptional activity and subcellular distribution under hypoxia

**Fig. 6:**
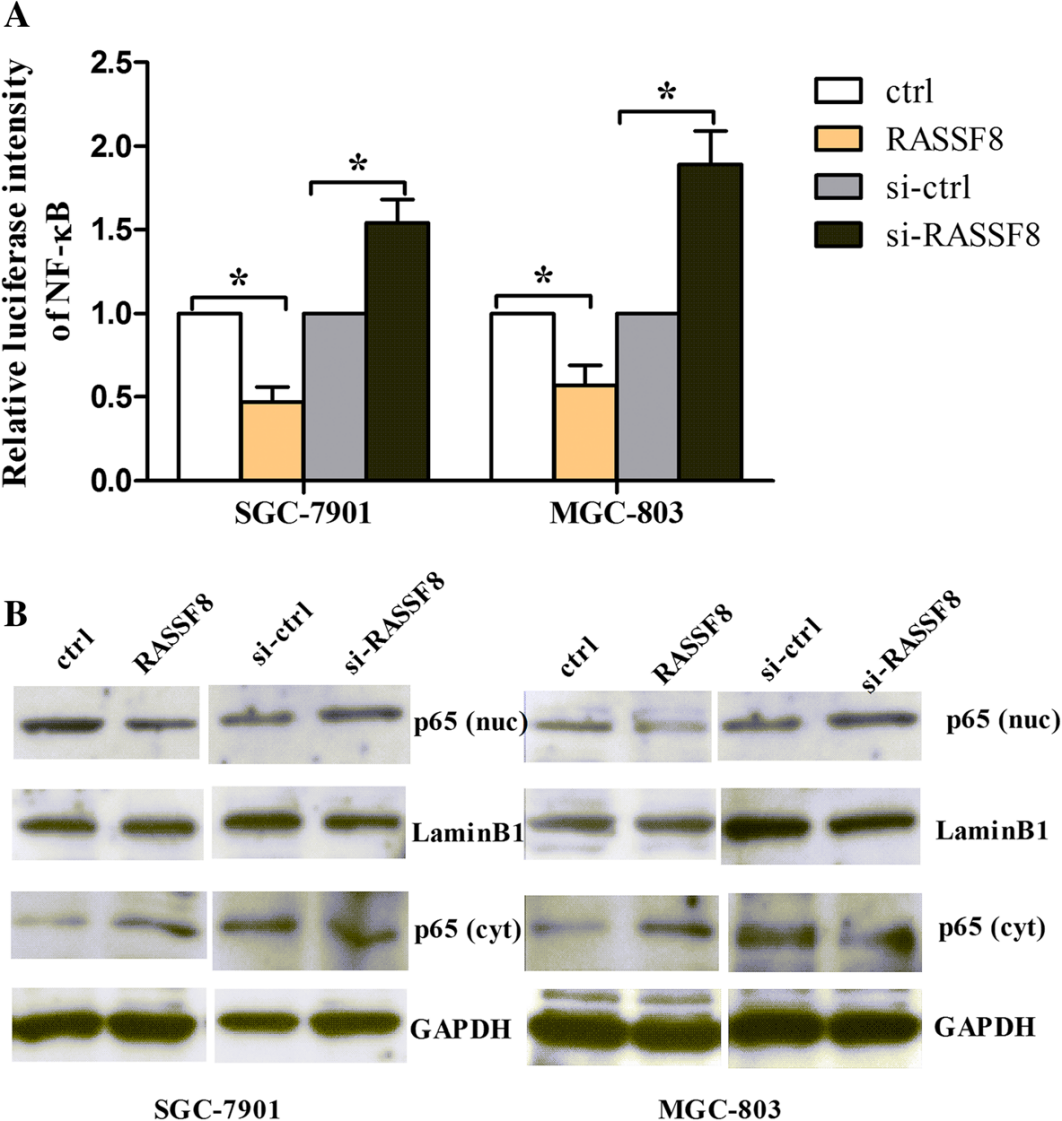
RASSF8 modulates the NF-κB transcriptional activity and p65 expression. **a** The cells were co-transfected with a NF-κB luciferase reporter plasmid and RASSF8 overexpressing plasmid or RASSF8 siRNA, and subjected to luciferase assay to detect the transcriptional activity of NF-κB. *P < 0.05. **b** The cells transfected with RASSF8-overexpressing plasmid or RASSF8 siRNA were exposed to Western blot to determine the expression of nuclear p65 and cytoplasmic p65 expression levels. LaminB1 was used as an internal control to normalize nuclear p65 expression and GAPDH was used as an internal control to normalize cytoplasmic p65 expression

Considering that RASSF8 is a negative regulator of NF-κB transcription activity in lung cancer [19], we aimed to examine NF-κB transcriptional activity with a NF-κB luciferase reporter plasmid in MGC-803 and SGC-7901 cells treated with RASSF8-overexpressing plasmid or RASSF8 siRNA under hypoxia. As shown in Fig. 6a, we found that RASSF8 overexpression reduced the NF-κB luciferase intensity, while RASSF8 knockdown increased the intensity. We then detected the subcellular distribution of NF-κB p65 in MGC-803 and SGC-7901 cells using Western blot. As expected, we found that RASSF8 knockdown increased the nuclear p65 expression, while RASSF8 overexpression attenuated it (Fig. 6b), suggesting that RASSF8 knockdown contributed to NF-κB transcriptional activity via the translocation of p65 from cytoplasm to nucleus.

### Selective inhibition of NF-κB by PDTC attenuates RASSF8 knockdown-induced cell growth and invasion

**Fig. 7:**
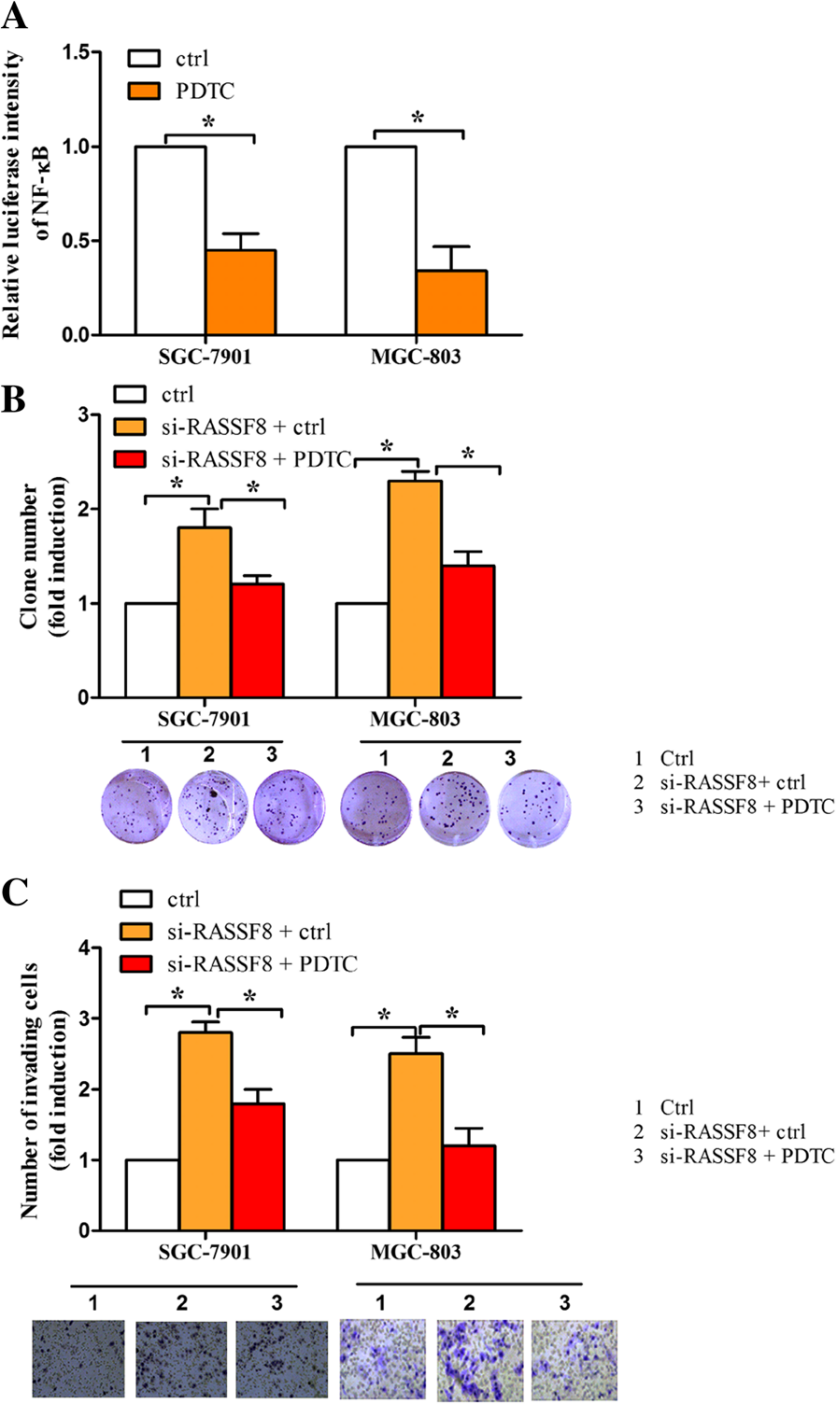
Inhibition of NF-κB by PDTC attenuates RASSF8 knockdown-induced cell growth and invasion. **a** NF-κB transcriptional activity was determined by luciferase assay in cells treated with PDTC or control. **b**–**c** SGC-7901 and MGC-803 cells transfected with RASSF8 siRNA or control were treated with or without PDTC, and then subjected to colony formation assay (**b**) and cell invasion assay (**c**). *P < 0.05. **d** Schematic representation of the regulatory mechanism of miR-224 in cell growth, migration and invasion in gastric cancer cells under hypoxia. Hypoxia induced HIF-1α expression, which can upregulated miR-224 at the transcriptional level. MiR-224 enhanced cell growth, migration and invasion through suppressing RASSF8, which was a direct target of miR-224. RASSF8 can modulate NF-κB transcriptional activity, which can reversely influence HIF-1α expression

Finally, we used a specific NF-κB inhibitor, pyrrolidine dithiocar bamate (PDTC), to inhibit NF-κB activity. NF-κB transcriptional activity was confirmed using a NF-κB luciferase reporter plasmid (Fig. 7a). The data from luciferase assay showed that PDTC significantly suppressed NF-κB transcriptional activity, suggesting that PDTC was effective. MGC-803 and SGC-7901 cells were treated with RASSF8 siRNA or together with PDTC, and subjected to colony formation and invasion assays under hypoxia. As shown in Fig. 7b, we found that PDTC attenuated the number of colonies induced by RASSF8 siRNA. Similarly, RASSF8 knockdown-induced cell invasion (Fig. 7c) was ameliorated when treated with PDTC compared to cells with control.

### MiR-224 and RASSF8 are inversely expressed in human gastric cancer specimens

**Fig. 8:**
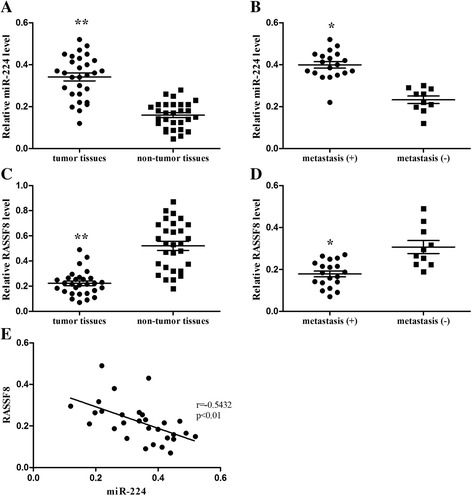
The expression levels of miR-224 and RASSF8 in gastric cancer specimens between gastric cancer tissues and adjacent non-tumor tissues (**a**, **c**), as well as lymph node metastasis positive- and negative- cancer tissues (**b**, **d**). **e** MiR-224 and RASSF8 expression levels were negatively correlated. *P < 0.05, **P < 0.01

We finally detected the expression levels of miR-224 using real-time PCR in gastric cancer tissues. As shown in Fig. 8a, we found that miR-224 was highly expressed in gastric cancer tissues compared to adjacent non-tumor tissues. Moreover, compared to gastric cancer tissues without lymph node metastasis, we found that miR-224 was upregulated in lymph node metastasis positive tissues (Fig. 8b). Inverse to the expression of miR-224, we found that RASSF8 was downregulated in gastric cancer tissues (Fig. 8c), as well as gastric cancer tissues with lymph node metastasis (Fig. 8d). As shown in Fig. 8e, we found that miR-224 and RASSF8 expression levels were negatively correlated. These data indicate that miR-224 and RASSF8 are related to the aggressiveness of gastric cancer.

## Discussion

In this study, we found that miR-224 and HIF-α were upregulated under hypoxia. HIF-1α upregulated miR-224 expression at the transcriptional level. MiR-224 was involved in hypoxia-induced cell growth, migration and invasion. We validated that RASSF8 was a direct target of miR-224 and mediated the effects of miR-224 under hypoxia. In addition, RASSF8 knockdown contributed to NF-κB transcriptional activity and subcellular redistribution. MiR-224 and RASSF8 were inversely expressed in human gastric cancer tissues and related to the aggressiveness of gastric cancer.

Accumulating evidence demonstrates that miR-224 plays important roles in the pathogenesis of diverse cancers. MiR-224 is upregulated and acts as an oncogene in non-small cell lung cancer [20, 21]. MiR-224 is also upregulated in esophageal squamous cell carcinoma and promotes cell proliferation, migration and invasion, and suppresses cell apoptosis, functioning as an oncogenic miRNA [22]. It has been reported that miR-224 enhances cell proliferation and suppresses cell apoptosis in meningioma cells by targeting ERG2 [23]. Previous studies have shown that miR-224 is induced under hypoxic condition in melanoma [13] and primary human trophoblasts [14]. Hypoxia has been validated to modulate miRNAs’ expression and plays important roles in miRNA functions [24]. In line with the findings in the above studies, we found that miR-224 expression was increased by hypoxia. In vitro and in vivo studies demonstrated that miR-224 promoted cell growth, while miR-224 inhibition suppressed cell growth induced by hypoxia. MiR-224 also promoted cell migration and invasion, and inhibition of miR-224 resulted in a decrease in cell migration and invasion induced by hypoxia.

HIF-1α, a crucial transcription factor, is known to regulate several hypoxia-related miRNAs [25]. Huang et. al has shown that HIF-1α regulates miR-210 expression through binding to the hypoxia-responsive element (HRE) in the region of miR-210 promoter in tumor initiation [26, 27]. HIF-1α can also regulate miR-155 expression via the HRE in miR-155 promoter during the prolonged hypoxia [28]. In agree with the mechanism of the regulation of miR-210 and miR-155 by HIF-1α, our study validated that HIF-1α upregulated miR-224 expression by binding to the HRE in the region of miR-224 promoter. We found that hypoxia increased the promoter activity of miR-224 and silencing of HIF-1α reduced the miR-224 promoter activity by luciferase assay. Moreover, when the mutations within the HRE were generated, the effects of hypoxia or HIF-1α on miR-224 promoter activity disappeared. Furthermore, ChIP assay showed that DNA fragment precipitated with anti-HIF-1α antibody generated a specific PCR product flanking the HRE.

RASSF8 is one of the members of RAS association domain family and is ubiquitously expressed in all major organs and tissues. The RASS family contains the classical RASSF proteins (RASSF1-6) and the four recently added N-terminal proteins (FASSF7-10) that are linked to biological processes, including cell proliferation, death, and response to hypoxia [29]. In our study, we validated that RASSF8 was a target of miR-224. RASSF8 was downregulated under hypoxia, opposite to that of miR-224. MiR-224 suppressed RASSF8 protein levels and the luciferase intensity controlled by RASSF8 3′UTR, while mutations within the binding sites between miR-224 and RASSF8 3′UTR abrogated the inhibitory roles of miR-224 in RASSF8 3′UTR. MiR-224 and RASSF8 expression levels were inversely expressed in gastric cancer. Several studies indicate that RASSF8 functions as a tumor suppressor in diverse cancers. In lung cancer, RASSF8 knockdown contributes to cell migration and invasion, acting as a tumor suppressor [19, 30]. RASSF8 downregulation enhances lymphangiogenesis and metastasis in esophageal squamous cell carcinoma (ESCC) and inversely correlates with patients’ survival. RASSF8 knockdown also enhances the expression of nuclear NF-κB p65, and NF-κB transcriptional activity in ESCC [31]. In cutaneous melanoma, RASSF8 knockdown promotes the cell growth, migration and invasion by increasing the expression of NF-κB p65 [32]. In agreement with the findings in the previous studies, our study found that RASSF8 overexpression suppressed the cell growth and invasion, while RASSF8 knockdown enhanced the cell growth and invasion that were inhibited by miR-224 inhibitor under hypoxia. In addition, we also found that RASSF8 overexpression inhibited the NF-κB transcriptional activity and nuclear p65 expression, while RASSF8 downregulation contributed to NF-κB transcriptional activity and p65 expression in nucleus. Moreover, inhibition of NF-κB by a specific inhibitor PDTC suppressed the cell growth and invasion that were induced by RASSF8 knockdown. A previous study also shows that hypoxia can activate NF-κB activation that is required for organism survival under hypoxia [33]. In hypoxic hepatocarcinoma cells, hypoxia can activate NF-κB, and NF-κB p65 and p50 could bind to HIF-1α promoter to increase its transcription [34]. In prostate cancer, inhibition of estrogen receptor β or hypoxia can stabilize HIF-1α which can contribute to the transcription of IKKβ, resulting in the activation of NF-κB [35]. In in endometrial carcinoma cells, hypoxia can activate NF-κB pathway, resulting in the transactivation of HIF-1α gene, while HIF-1α can enhance NF-κB transcriptional activity [36]. The above positive feedback loop between NF-κB and HIF-1α facilitates the tumor adaptation to microenvironmental hypoxia in cancer cells. Therefore, we propose that NF-κB may enhance HIF-1α expression in gastric cancer cells under hypoxia, which needs further studies.

## Conclusion

We have demonstrated that miR-224 was upregulated under hypoxia and regulated by HIF-1α at the transcription level. MiR-224 promotes the cell growth, migration and invasion through the downregulation of RASSF8 under hypoxia, which was in line with the results reported in the recent article that miR-224 promotes the progression of cervical cancer by directly RASSF8 [37]. RASSF8 knockdown can activate the NF-κB transcriptional activity and subcellular distribution (Fig. 7d). The study suggest that inhibition of miR-224 may serve as a prospective therapeutic target for hypoxic gastric cancer. Although there is still a long way to go before the advent of miRNA-based cancer therapy, inhibiting miR-224 might provide a new approach for gastric cancer treatment in clinic in future.

## Abbreviations

3′UTR: 3′untranslated region
ChIP: Chromatin immunoprecipitation
HIF-1α: hypoxia-inducible factor-1α
miRNA: microRNA
MTT: 3-(4,5-dimethyl-2-thiazolyl)-2,5-diphenyl-2-H-tetrazolium bromide
RASSF8: RAS association domain family member 8
